# Gastric Ischemia after SARS-CoV-2 Infection

**DOI:** 10.3390/life14010047

**Published:** 2023-12-27

**Authors:** Woo Yong Lee, Byung Woo Yoon

**Affiliations:** 1 Department of Surgery, Inje University Haeundae Paik Hospital, 875 Haeundae-ro, Haeundae-gu, Busan 48108, Republic of Korea; yongaaa5972@naver.com; 2 Department of Internal Medicine, Chungang University Gwangmyung Hospital, Dukanro 110, Gwangmyung 14353, Republic of Korea

**Keywords:** COVID-19, gastric ischemia, gastric pneumatosis

## Abstract

Gastric ischemia is uncommon because the stomach has multiple collateral blood supplies. The etiology of gastric ischemia is vascular insufficiency caused by systemic hypotension, vasculitis, or disseminated thromboembolism. Mechanical causes include gastric volvulus and acute gastric distention. Uncommon as gastric ischemia is, we are the first to report a 65-year-old male who developed gastric ischemia leading to gastric pneumatosis 26 days after initial treatment for severe acute respiratory syndrome coronavirus-2 infection (SARS-CoV-2), via laparoscopic imaging. We conclude that physicians should be suspicious of gastric ischemia when the patient is infected with SARS-CoV-2 with severe abdominal pain and should proceed with medical conservative care instead of surgery.

## 1. Introduction

Gastric ischemia (GaI) is a rare condition because the stomach has multiple collateral blood supplies and is, therefore, less prone to ischemia [1]. However, GaI can become a serious disease when it is located between the esophagus and duodenum [1]. Similar to other gastrointestinal disorders, the symptoms of GaI include abdominal pain, nausea, vomiting, and bleeding, as seen in other gastrointestinal disorders [2]. The known causes of GaI include sepsis, severe atherosclerosis, vasculitis, acute gastric distension, and gastric volvulus [1].

According to recent case reports, severe acute respiratory syndrome coronavirus 2 (SARS-CoV-2) infection may cause acute gastrointestinal ischemia, especially in the small bowel and colon, with the onset of ischemia occurring 1 to 13 days after SARS-CoV-2 infection [3,4]. The cause of gastrointestinal ischemia is multifactorial, involving inflammation, microvascular endotheliopathy, and a hypercoagulable state that leads to tissue hypoxia related to SARS-CoV-2 infection. Clinical data have shown that coronavirus disease (COVID-19) is associated with a significant risk of thrombotic complications, including microvascular thrombosis, venous thrombosis, and stroke [5]. Recently, it was shown that there is a correlation between elevated acute-phase reactants, such as fibrinogen and C-reactive protein (CRP), which may contribute to COVID-associated hypercoagulability [6,7,8]. SARS-CoV-2 infects via angiotensin-converting enzyme 2 (ACE2) receptors, which are thought to be abundant in affected organs and tissues, such as the oral mucosa, esophagus, small intestine, colon, liver, spleen, and the respiratory system. In addition, ACE2 activation stimulates the excessive release of Von Willebrand factor, factor VIII, and plasminogen activator-1 by endothelial cells. This can lead to inflammation, tissue injury, and ischemia [9]. As a result, ischemia occurs in organs that do not have a rich blood supply or collaterals; thus, the stomach is less likely to experience ischemia. We report an unusual case of GaI, despite the stomach having a rich collateral blood supply and fewer ACE2 receptors [10]. To the best of our knowledge, this is the first reported case of GaI following SARS-CoV-2 infection. 

## 2. Case Presentation 

A 65-year-old man with a history of chronic kidney disease and diabetes mellitus presented to the emergency room with high fever, cough, and general weakness. Physical examination revealed blood pressure of 110/70 mmHg, heart rate of 90/min, respiratory rate of 23/min, and tympanic temperature of 39.2 °C with bilateral basal crackles. 

His initial laboratory work-up was WBC 15,300 (reference: 5000–10,000/mm^3^), hemoglobin 9.5 (reference: 12–16 g/dL), platelet 198,000 (reference: 140,000–400,000/mm^3^), CRP 120 (reference: <5 mg/dL), D-dimer 4.13 (reference: <0.5 mcg/mL), CK-MB 20.4 (reference: <4.0 ng/mL), and hs troponin-I 28.2 (reference: <15.6 pg/mL). Owing to elevated cardiac enzyme levels, a cardiac echography was performed, which showed no abnormalities. His initial baseline blood uric nitrogen (BUN) level was 35 mg/dL (reference: 10–26 mg/dL) and creatinine was 2.3 (reference: <1.5 mg/dL); however, his admission blood urea nitrogen (BUN) level was 85 mg/dL and creatinine was 4.4 mg/dL.

**Figure 1:**
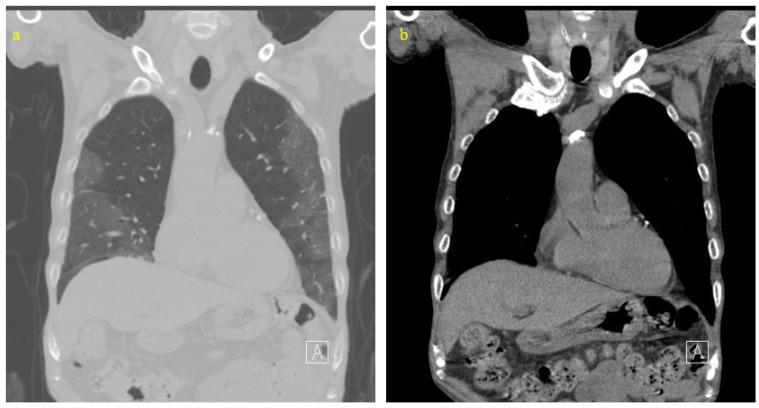
Initial chest computerized tomography: lung mode (**a**) and bone mode (**b**). Diffuse glass-ground opacities can be seen in the left and right areas, indicating viral pneumonia, which was SARS-CoV-2 infection (**b**), and no definite infection focus in the gastrointestinal organs (**b**).

Initial chest computed tomography (CT) showed typical severe acute respiratory syndrome coronavirus 2 (SARS-CoV-2) pneumonia patterns with diffuse ground-glass opacities in both lungs (Figure 1a). No gastric abnormalities were observed (Figure 1b). His SARS-CoV-2 via real time-polymerase chain reaction test confirmed positive, which led to his admission. We started treatment with 30 mg of methylprednisolone for five days and 10 mg of mPD for an additional five days, 200 mg of loading dose and a maintenance dose of 100 mg/day of remdesivir, and 400 mg/day of moxifloxacin for five days. Upon admission, his initial SARS-CoV-2 symptoms of fever, cough, and tachypnea improved with remdesivir and steroid treatment.

However, the patient developed metabolic acidosis with an arterial blood gas (ABG) result of pH 7.22, pCO_2_ 32 (reference: 32–45 mmHg), pO_2_ 92 (reference: 83–108 mmHg), and HCO_3_^−^ 12.6 (reference: 21–28 mmol/L), which led to continuous renal replacement therapy for three days starting on the 8th day of hospitalization and hemodialysis for four days. Hemodialysis was discontinued after the 15th day of hospitalization, when his ABG normalized to pH 7.38, pCO_2_ 38, pO_2_ 100, and HCO_3_^−^ 23.8.

**Figure 2:**
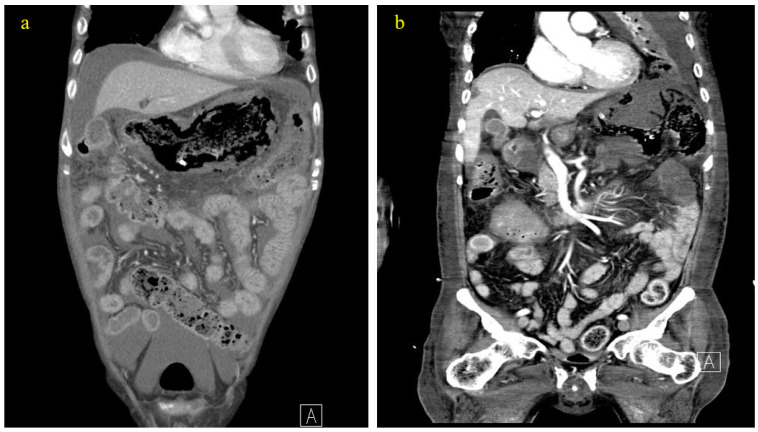
Abdominal computerized tomography (CT) show air around the stomach with ascites on the 20th hospital day (**a**), indicating gastric perforation. The post-operational CT shows an increased extent of ischemia/infarction in multiorgan in the abdomen, including small bowel, colon, liver, pancreas, and spleen (**b**).

**Figure 3:**
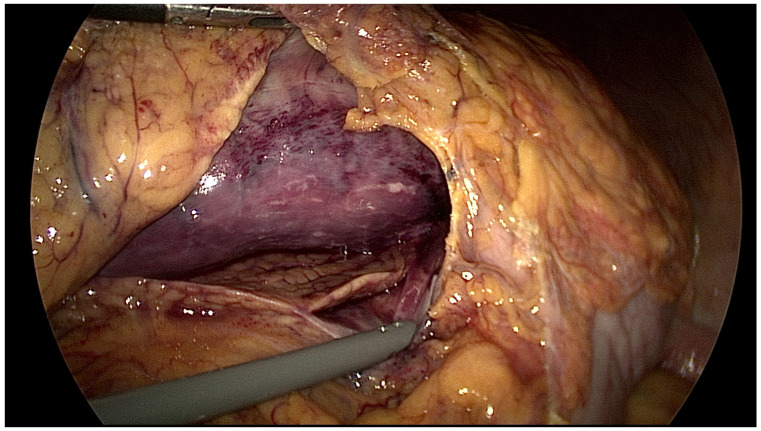
Laparoscopic view of the stomach. The stomach is “blue”, indicating low blood perfusion or gastric ischemia. The wall is edematous and a small amount of bloody ascites can be seen. There is no tissue debris, necrosis or liquefaction of the stomach indicating gastric necrosis.

On the 16th day of hospitalization, the patient presented with a fever of 38.3 °C. CRP increased from 35 to 90 mg/dL, so tazoperan/pieracillin 2.25 g every 6 h was started. However, on the 20th day of hospitalization, the patient experienced excruciating abdominal pain, and a physical examination revealed rebound tenderness in the upper abdomen, for which abdominal CT was performed. Computed tomography (CT) revealed gastric pneumatosis with diffuse intramural gas around the stomach wall (Figure 2a), suggesting minimal gastric perforation. A consult for gastric endoscopy was requested; however, gastric endoscopy was not recommended by the gastrologist as this may push more air into the abdomen, and the patient was referred for surgery and an emergency laparoscopic exploration was performed on the 21st hospital day. The stomach was edematous with a “blue” color change, indicating low blood perfusion or gastric ischemia in Figure 3. Additionally, a moderate amount of bloody ascites filled the abdomen, indicating bleeding. However, a thorough examination with a nasogastric tube and gas insertion was performed, and there was no air leak, indicating no signs of perforation in the gastric wall. Furthermore, there were no findings of perforation in the small bowel or colon, and no bleeding within the laparoscopic field of view. Finally, there was no tissue debris, necrosis or liquefaction around the stomach indicating gastric necrosis. Based on the CT and surgical findings, the patient was diagnosed with gastric ischemia by the attending surgeon. Surgery was completed after drain insertion and aspiration of the ascites.

**Figure 4:**
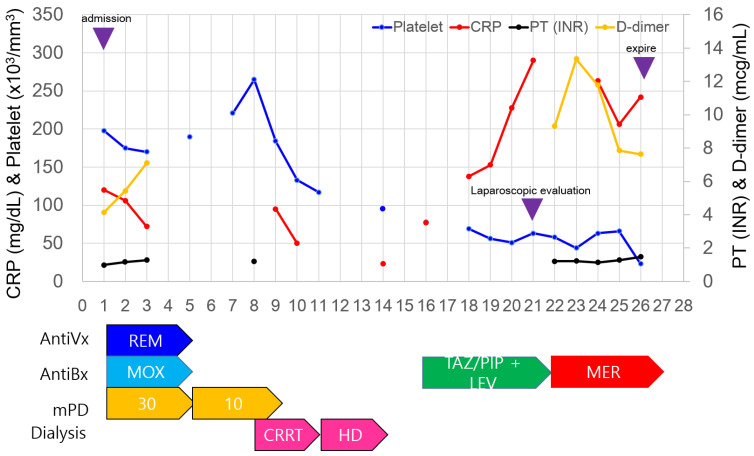
The timeline of the patient’s treatment and laboratory findings. On the left side of the y-axis is C-reactive protein (CRP) and platelet count, and on the right side of the y-axis is prothrombin time (PT) and D-dimer. The patient was on remdesivir (REM) for five days, and moxifloxacin (MOX) for 5 days for SARS-CoV-2 infection treatment with methylprednisone (mPD) 5 mg/day for five days and 10 mg/day for five days. After treatment of MOX, he was on tazoperan/piperacillin (TAZ/PIP) and levofloxacin (LEV). After surgery, he was on meropenem (MER). Purple arrows indicate hospital day of admission, laparoscopic evaluation, and expiration. The patient was on continuous renal replacement therapy (CRRT) for three days and hemodialysis (HD) for four days.

Postoperative blood tests were unremarkable, and conservative treatment with total parental nutrition, protein pump inhibitors, and intravenous meropenem of 0.5 mg was administered every 12 h due to increased CRP. Abdominal CT performed on the third day after surgery (24th hospital day) showed increased ischemia/infarction in multiple organs of the abdomen, including the stomach, small bowel, colon, liver, pancreas, and spleen (Figure 2b). The patient died of multiple organ failure on the 26th hospital day. The timeline for diagnosis and treatment are summarized in Figure 4. 

## 3. Discussion

GaI is a rare disease that has mainly been reported in the literature as case reports. GaI is very rare as the stomach has abundant blood supplies compared with other intra-abdominal organs, which hinders the stomach from experiencing sudden ischemic events [1]. Thus, the previously reported GaI was thought to be caused by vascular insufficiency due to mechanical pressure or underlying anatomical abnormalities of the stomach. 

GaI may occur partially or entirely according to the obstruction of blood flow. Regardless of severity, GaI can be fatal, requiring emergency surgery or even leading to death. The underlying mechanisms of GaI are mucosal edema, vascular congestion, and superficial necrosis, which leads to full-thickness hemorrhagic necrosis with deep ulceration of the gastric wall [1]. Symptoms of GaI are abdominal pain, nausea, vomiting, upper gastrointestinal bleeding, and abdominal distension-like acute abdomen [2]. Thus, GaI may be underdiagnosed or misdiagnosed from the observation of symptoms. Regardless of severity, GaI can be fatal, requiring emergency surgery or even leading to death by peritonitis and septic shock. 

Most recently, following the COVID-19 pandemic, several reviews of gastrointestinal ischemia have been reported, mostly in the small bowel or colon, with abdominal bleeding or hematoma on the abdominal wall [3,4,11,12,13,14]. The possible mechanisms of ischemia due to SARS-CoV-2 infection are multifold, involving inflammation, microvascular endotheliaopathy, and hypercoagulable state that leads to tissue hypoxia. 

The most well-known infection pathway of SARS-CoV-2 is via ACE2 receptors, which are abundant in the oral mucosa, esophagus, small intestine, colon, liver, spleen and the respiratory system [10]. However, the stomach has fewer ACE2 receptors than these organs [10]. Thus, SARS-CoV-2 may not have directly caused GaI via ACE2 receptors in our case. 

When considering hypercoagulable state due to SARS-CoV-2, the underlying pathway is multifactorial: cytokine storm, complement pathway, autoimmunity, ACE2 expression, endothelial damage, and fibrinolysis shutdown may be in play [9]. However, the patient in our case did not experience acute initial shock symptoms related to cytokine storm, and he had GaI on the 20th hospital day, which may be considered as a delayed reaction, suggesting endothelial damage or fibrinolysis shutdown to be the main trigger.

Several methods are available for diagnosing GaI. First, CT can detect air in the portal vein or gastric pneumatosis. This is the most common method of diagnosing GaI. However, ischemia caused by COVID-19 differs from ischemia from other causes, as COVID-19-related ischemia usually involves thrombosis in the small vessels [11,15]. Secondly, gastroscopy is used to reveal mucosal injury to the lining of the stomach. Finally, an operation may be performed, which is seldom done for diagnosis only and is reserved only if surgery can resolve mechanical problems leading to GaI, such as volvulus. 

Differential diagnosis with GaI as in our case is gastric necrosis and gastric perforation. The CT images may be similar with gastric necrosis and gastric perforation [16,17]. However, the differential diagnosis can be done easily in the operating room where gastric necrosis has loss of structural integrity, i.e., the organ cannot recover, which can be the endpoint of ischemia, and air leak in perforation. Upon examination, we considered our patient GaI as there was no gastric distension, gastric debris, black color change, omental liquefaction or hemorrhagic fluid collection, which are all signs of gastric necrosis. Gross pathologic images from case reports of the stomach when gastric necrosis occurs is different from our case of GaI [16,17]. In terms of treatment, if the patient has gastric necrosis, gastrectomy should be the main treatment option to remove dead tissue. In gastric perforation, if it is a micro-perforation, an omental patch or wedge resection can be done, but if it is severe, primary repair should be performed. 

This case is unique for several reasons. Firstly, we present GaI after SARS-CoV-2 infection. Secondly, GaI occurs in the large vessels surrounding the stomach, instead of in organs with small vessels. Although there was no thrombosis around the vessels involved in the stomach, the stomach wall was thickened, indirectly indicating hypoperfusion of the stomach vessels. Thirdly, despite having fewer ACE2 receptors than other organs, the stomach had severe ischemia compared to organs with abundant ACE2 receptors. Fourthly, GaI was not observed during surgical procedures before this case, especially after the COVID-19 infection. 

In retrospect, we have regrets about the treatment of the patent. The main treatment for GaI is supportive care with fluid resuscitation, intravenous antibiotics, nasogastric tube decompression, and gastric acid suppression. In our case, we performed the operation as the reading of the abdominal CT showed that there was a perforation in the stomach, which is the standard treatment of gastric perforation. However, surgery is contraindicated in GaI unless there is a volvulus or hiatal hernia, because the prognosis is very poor. Thus, after determining that there was no perforation after a thorough air leak test using a nasogastric tube, we concluded that supportive care should be provided instead of surgery. Furthermore, we speculate that, although GaI is highly unlikely to occur, it may be more severe in patients recently infected with SARS-CoV-2 with underlying comorbidities. Similar but different cases of emphysematous gastritis have been reported, especially in patients with CKD or hemodialysis patients [18]. This may be due to the fact that vasculopathies may have developed in CKD and hemodialysis patients, making them more prone to mesenteric ischemia [19]. The infection of SARS-CoV-2 may have been the final trigger in CKD or hemodialysis patients; however, when considering the total CKD or hemodialysis patients on a global population scale, more documented cases are needed to conclude that CKD or hemodialysis patients have a higher risk of GaI. Another speculation is steroid use with remdesivir, which may have caused undetected micro-perforation and healing or coagulopathies before the main event. Although the patient was on low dose steroids, and the duration was a short term of ten days, this may have triggered transient hyper-coagulopathies or other reduced mucosal barrier mechanisms which may have caused GaI [20,21]. 

In terms of future research, we regret that an autopsy and a sample of the stomach were not performed. A biopsy of the stomach with molecular markers for ACE2 receptors may have revealed if this patient had more ACE2 than other patients, leading to GaI from SARS-CoV-2. Furthermore, an autopsy may have revealed the direct and indirect cause of GaI, from the start of SARS-CoV-2 infection. However, due to Confucian culture in Korea, it was hard to request to perform a biopsy of the patient without legal proceedings [22]. Future cases should include biopsies to reveal the causal relationship between SARS-CoV-2 and GaI. 

## 4. Conclusions

Our conclusions are two-fold. First, physicians should maintain a high level of suspicion for GaI if the patient has an infection with SARS-CoV-2 virus, with symptoms of severe abdominal pain. Second, in these cases, conservative treatment should be prioritized over immediate surgery, and if necessary, minimal surgical intervention should be considered. 

## Data Availability

Data are contained within the article.
